# Molecular Features of Non-Selective Small Molecule Antagonists of the Bradykinin Receptors

**DOI:** 10.3390/ph13090259

**Published:** 2020-09-21

**Authors:** Bahareh Rasaeifar, Patricia Gomez-Gutierrez, Juan J. Perez

**Affiliations:** Department of Chemical Engineering, Universitat Politecnica de Catalunya. ETSEIB. Av. Diagonal, 647, 08028 Barcelona, Spain; bahareh.rasaeifar@upc.edu (B.R.); ondopasa@gmail.com (P.G.-G.)

**Keywords:** bradykinin receptors, bradykinin pharmacophore, non-peptide bradykinin antagonists, bradykinin repurposing, Covid-19 therapy

## Abstract

Angiotensin converting enzyme 2 (ACE2) downregulation is a key negative factor for the severity of lung edema and acute lung failure observed in patients infected with SARS-CoV-2. ACE2 downregulation affects the levels of diverse peptide mediators of the renin-agiotensin-aldestosterone and kallikrein-kinin systems, compromising vascular hemostasis. Increasing evidence suggests that the inflammatory response observed in covid-19 patients is initiated by the action of kinins on the bradykinin receptors. Accordingly, the use of bradykinin antagonists should be considered as a strategy for therapeutic intervention against covid-19 illness progression. Presently, icatibant is the only bradykinin antagonist drug approved. In the present report, we investigated the molecular features characterizing non-selective antagonists targeting the bradykinin receptors and carried out a in silico screening of approved drugs, aimed at the identification of compounds with a non-selective bradykinin antagonist profile that can be evaluated for drug repurposing. The study permitted to identify eight compounds as prospective non-selective antagonists of the bradykinin receptors, including raloxifene; sildenafil; cefepime; cefpirome; imatinib; ponatinib; abemaciclib and entrectinib.

## 1. Introduction

Beginning in December 2019, a novel coronavirus designated SARS-CoV-2 was identified as the pathogen causing an international outbreak of respiratory illness termed Covid-19, originated in Wuhan, Hubei Province, China. Despite the virus has a fatality rate of only ~2–3% it exhibits a high transmission rates, resulting in a high overall death toll that forced the World Health Organization to declare SARS-CoV-2 as a pandemic infectious disease of international concern on 11 March 2020 [1]. Until 18 July 2020, there have been 14,108,240 confirmed cases of Covid-19 with 602,695 confirmed deaths [2]. Unfortunately, ~20% of the people infected are likely to develop pneumonia of varying severity that may evolve to acute respiratory distress syndrome (ARDS), sepsis, and death [3]. Presently, clinical treatment of Covid-19 is mainly symptomatic by using anti-inflammatories like dexamethasone [4] or cytokine inhibitors, combined with antibiotics to treat secondary infections. Knowledge of the mechanism behind SARS-CoV-2 infection will help to identify other therapeutic agents to be used for the treatment of patients with Covid-19. This report focuses in the mechanism of infection and the implication of the renin-angiotensin-aldosterone and the kallikrein/kinin systems in illness progression [5].

**Figure 1:**
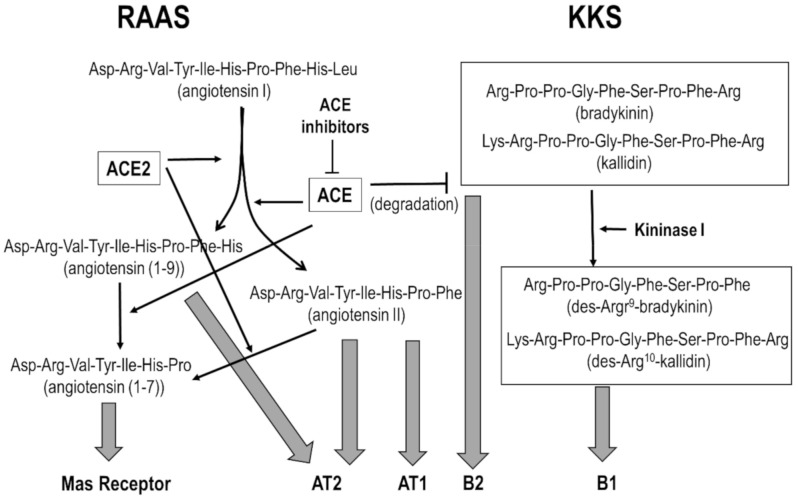
Crosstalk between RAAS and KKS. The angiotensin converting enzymes ACE and ACE2 are key players of RAAS, regulating the production of diverse mediators (see text), producing a plethora of physiological actions through the activation of different receptors (solid arrows). Thus, activation of the angiotensin AT1 receptor produces vasoconstriction, hypertrophy and fibrosis; whereas activation of the AT2 and Mas receptors produce vasodilation, antihypertrophy and antifibrosis. On the other hand, ACE regulates the levels of kinins that produce vasodilatation and increased vascular permeability through the B1 and B2 receptors.

The renin-angiotensin-aldosterone system (RAAS) [6] and the kallikrein/kinin system (KKS) [7] are involved in the regulation of intravascular volume, blood pressure and tissue repair via inflammatory and proliferative mechanisms. The angiotensin converting enzyme (ACE) and the angiotensin converting enzyme 2 (ACE2) are key players in both systems. Cross-talk between the two systems is summarized in Figure 1. ACE is a carboxydipeptidase that produces the octapeptide angiotensin II from its inactive precursor angiotensin I, orchestrating a plethora of actions including sodium reabsorption and increase of blood pressure mediated through the AT1 receptor and vasodilation and natriuresis mediated through the AT2 receptor [8]. On the other hand, ACE2 is an integral membrane carboxypeptidase with its catalytic domain at the extracellular side that counterbalances the actions of ACE. Specifically, ACE2 degrades angiotensin II to produce angiotensin (1–7), a peptide that elicits vasodilation and natriuresis via activation of the Mas receptor [9]. Furthermore, ACE2 also converts angiotensin I into angiotensin (1–9), a peptide that elicits vasodilation and anti-inflammatory effects through activation of the AT2 receptor [10]. Angiotensin (1–9) is further converted into angiotensin (1–7) by the action of ACE [8]. On the other hand, kallikreins are serine proteases that produce bradykinin (BK) and kallidin (Lys-BK) -two members of the kinin family- from kininogens in response to inflammation, trauma, burns, shock, allergy and some cardiovascular diseases [11]. Other members of the kinin family include the corresponding des-Arg analogs: des-Arg^9^-BK and des-Arg^10^-Lys-BK.

Kinins produce a plethora of physiological actions including vasodilatation and increased vascular permeability via activation of the B1 and B2 receptors [12,13]. The former is upregulated during inflammation episodes or tissue trauma, whereas the latter is constitutively expressed in a variety of cell types. BK and Lys-BK are agonists of the B2 receptor, whereas the des-Arg analogs are agonists of the B1 receptor [14]. ACE and the ACE2 enzymes are actors involved in the cross-talk between RAAS and KKS. The former upregulates angiotensin II and downregulates BK, whereas the latter upregulates angiotensin (1–9) and downregulates des-Arg kinins, respectively [15].

SARS-CoV-2 binds with high affinity to ACE2 facilitating cell fusion and entry [16,17]. Endocytosis of the SARS-CoV-2 spike protein-ACE2 complex into endosomes reduces surface ACE2 expression, being detrimental for its role in tissue protection; producing a clear interference on RAAS mediated homeostasis functions. Taking into account that ACE2 is more abundantly present in the epithelia of the lungs and on lymphocytes [18], its downregulation is a key negative factor for severity of lung edema and acute lung failure observed in patients infected by SARS-CoV. Actually, downregulation of ACE2 translates into altered levels of diverse mediators of the SAARS and KKS. Specifically, levels of angiotensin II are increased and in turn, those of the des-Arg kinins due to a lower availability of ACE to degrade BK and Lys-BK, whereas levels of angiotensin (1–9) and angiotensin (1–7) are decreased. About fifteen years ago, it was hypothesized that the observed physiological effects produced in patients infected by SARS-CoV were due to the actions of angiotensin II on the AT1 and AT2 receptors [19,20]. Presently, there is growing evidence that inflammation may be triggered through the des-Arg peptides/B1 axis-mediated signaling pathway [21,22,23,24]. This new perspective suggests that inhibition of BK signaling may be a suitable therapy to avoid the cytokine storm associated with the Covid-19 illness [25].

**Figure 2:**
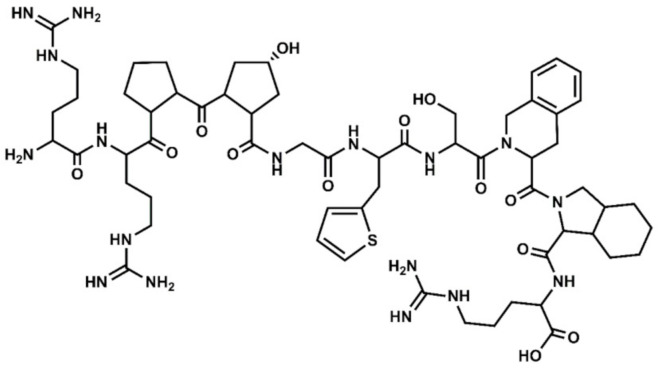
Chemical structure of icatibant, together with its residue sequence. Hyp=hydroxylproline; Thi = thiophenyl-alanine; Tic = 1,2,3,4-tetrahydroisoquinolin-2-ylcarbonyl; Oic = (3aS,7aS)-octahydroindol-2-ylcarbonyl.

Based on this novel mechanistic hypothesis, selective and non-selective BK antagonists should be considered as therapeutic agents for the treatment of covid-19 [26,27]. Despite the enormous effort devoted in the past to design peptide and non-peptide selective ligands targeting the BK receptors [13,28], icatibant (Figure 2) is the only BK antagonist presently approved as therapeutic agent for the symptomatic treatment of acute attacks of hereditary angioedema in adults with C1-esterase-inhibitor deficiency [29]. Despite being a B2 selective antagonist, the compound is presently involved in a clinical trial to assess its benefits for the treatment of the covid-19 illness [30]. Considering the lack of BK antagonists in the market and the urgency to have new treatments for the covid-19 available, drug repurposing is a valuable strategy for quickly discover novel therapeutic uses of already approved drugs. Specifically in this case, the discovery of approved therapeutic agents with a BK antagonist profile.

Virtual screening methods can be very valuable in drug repurposing, provided we count on specific structural knowledge of the therapeutic target of interest [31]. Specifically, for BK we recently reported the results of a modeling study addressed to analyze the stereochemical features required for non-peptide selective ligands to bind to the B1 and B2 receptors [32,33]. Furthermore, the results of the study also permitted to identify the stereochemical features associated with selective binding to each of them [34]. As a complement to that work, we discuss in the present contribution the characterization of the molecular features that confer a non-selective binding profile to small molecule ligands targeting the B1 and B2 receptors and show preliminary results from a virtual screening aimed at the identification of approved drugs with a non-selective profile to the BK receptors.

## 2. Results and Discussion

### 2.1. Stereochemical Features of Non-Selective Small Molecule Ligands Targeting the B1 and B2 Receptors

**Figure 3:**
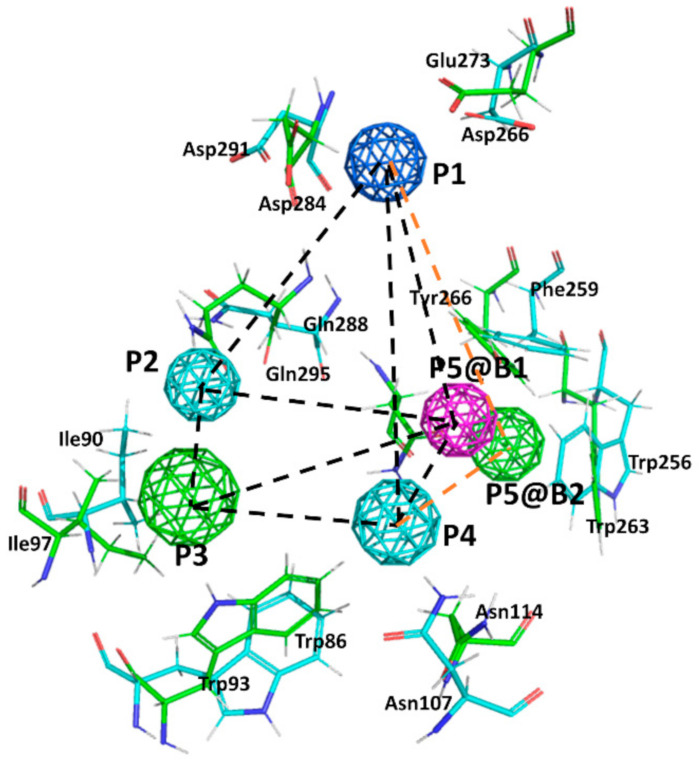
Superposition of the B1 and B2 receptor pharmacophores. Colored spheres represent pharmacophore points (P1–P5) according to the following color code: dark blue represents a positive charge moiety; magenta a hydrogen bond accepting center; light blue a hydrogen bond donor/acceptor center; green an aromatic/lipophilic center. @B1 and @B2 is used to differentiate P5 for the B1 and B2 receptors, respectively. Consensus distances between common pharmacophore points are (black dotted lines): d(1,2) = 9 Å; d(1,3) = 14 Å; d(1,4) = 10.5 Å; d(2,3) = 6 Å; d(2,4) = 7 Å; d(3,4) = 7.5 Å. Specific distances for the B1 pharmacophore: d(1,5) = 9.5 Å; d(2,5) = 9.3 Å; d(3,5) = 9.5 Å; d(4,5) = 5.7 Å; whereas for the B2 pharmacophore are (orange dotted lines): d(1,5) = 11 Å; d(2,5) = 9 Å; d(3,5) = 8.8 Å; d(4,5) = 8.4 Å. Side chains of the main residues involved in defining the binding pocket for non-peptide ligands are explicitly depicted: green for the B1 and blue for the B2, respectively.

Due to the lack of crystallographic structures of the BK receptors, the construction of 3D models at atomic resolution by homology modeling of the B1 and B2 receptors was recently performed and reported [32,33]. Models were subsequently used to undertake a docking study that permitted the analysis of diverse ligand-receptor complexes of known selective small molecule compounds. From this study, the corresponding pharmacophores describing the stereochemical features that ligands must fulfill for binding to each of the two receptors were defined. The two receptors share a high sequence identity of 28% (sequence homology is 43%) that reaches ~50% when the orthosteric sites are compared. Accordingly, it is expected to find common residues in their respective orthosteric sites. Comparison of the pharmacophores shows that they exhibit four points in common (Figure 3): a positive charge (P1); a hydrogen bond donor/acceptor (P2); an aromatic ring (P3); and a hydrogen bond donor/acceptor (P4). Point P5 in the two pharmacophores discriminates binding to the two receptors: in B1 is hydrogen bond acceptor, whereas in B2 is hydrophobic/aromatic moiety. Accordingly, ligands fulfilling pharmacophore points P1–P4, common to the two receptors are expected to be non-selective bradykinin antagonists. As a proof of concept, we disclosed a short list of non-selective hits identified through the virtual screening of a large database of small molecules [34]. In contrast, the design of selective ligands is trickier. In addition of the differential chemical nature of P5 in the two receptors, analysis of the 3D models of the ligand-receptor complexes suggests that there is a steric hindrance that prevents selective B1 ligands to bind B2 and, vice versa. The steric hindrance is produced by the differential nature of the side chains of the non-conserved residues Arg^202^ in B1 compared to its counterpart, Thr^197^ in B2 [34].

### 2.2. Drug Repurposing

**Figure 4:**
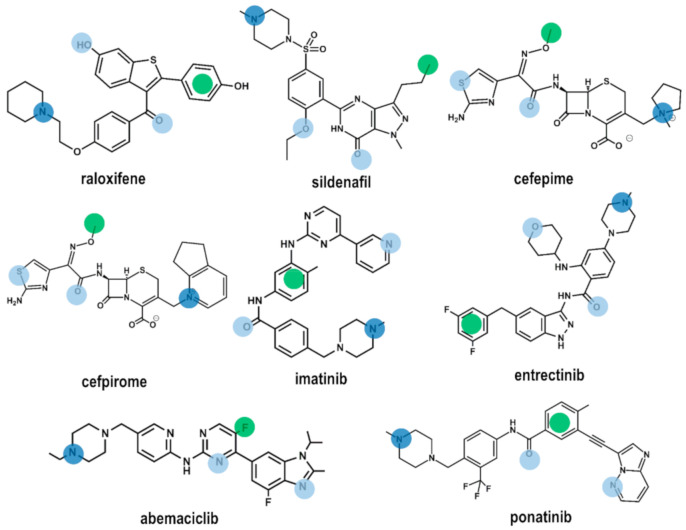
Chemical structures of the eight compounds identified by virtual screening of the DrugBank database. Color dots are drawn on top of the moieties responsible for fulfilling pharmacophore points P1–P4 according to the color code described in Figure 1.

Using the four common pharmacophore points P1–P4 as a query, we carried a in silico screening on the DrugBank using the Molecular Operating Environment (MOE) program [35]. The DrugBank is a database containing comprehensive information of all FDA approved drugs [36]. In order to carry out the virtual screening study, we first generated the corresponding 3D DrugBank database using the Database Viewer module of MOE [35]. The database contains for each molecule its 3D structure together with a set of conformations, generated using a build-up procedure from systematic conformational searches of molecular fragments. Virtual screening was carried out on a subset of 1703 molecules selected according to their molecular weight between 200 and 600. Hits obtained were subsequently docked onto the 3D models of the B1 and B2 receptors, respectively to check for possible steric hindrance. Preliminary results of the virtual screening yielded eight drugs (Figure 4): raloxifene [37], a selective estrogen receptor modulator; sildenafil [38], a phosphodiesterase type 5 inhibitor; cefepime [39] and cefpirome [40], two β-lactam antibiotics; imatinib [41] and ponatinib [42], two bcr-Abl tyrosine kinase inhibitors; abemaciclib [43], a dual inhibitor of cyclin-dependent kinases 4 and 6; and entrectinib [44] a non-selective tyrosine kinase inhibitor. According to the results of this study, these compounds exhibit the characteristics to be non-selective BK ligands. Figure 4 shows the location of the common pharmacophore points on their 2D chemical structures. Evaluation of the ability of these compounds to act as B1 and B2 receptor antagonists is currently underway.

Among the drugs identified, there are no studies reporting a direct BK antagonistic profile of any of them. However, in the case of raloxifene there are studies that show a synergistic action with bradykinin. Actually, rats treated with raloxifene show an increased reduction of systolic blood pressure on administration of bradykinin, suggesting an enhanced bioavailability of NO in these animals [45]. Sildenafil does not exhibit a synergetic action in the reduction of BK induced glucose uptake in humans when administered together with N^ω^-monomethyl-L-arginine a nitric-oxide synthase inhibitor [46], indirectly suggesting that sildenafil may not interact with the BK receptors.

## 3. Materials and Methods

The 3D models of the B1 and B2 were constructed by homology modeling using the chemokine CXCR4 receptor as template (pdb entry code 3ODU) [47], following the procedure explained elsewhere [32,33]. Initial crude models of the receptors were constructed by threading the sequences of the B1 and B2 receptors onto the backbone of the template following the sequence alignment and subsequently validated using the Modeller 9 version 8 (9v8) software [48]. Next, models were refined using molecular dynamics simulations using a system consisting of each of the respective receptors embedded in a lipid bilayer of 1-palmitoyl-2-oleoyl-sn-glycero-3-phosphocholine (POPC) lipids and water molecules, using GROMACS 4.6 package [49] as described elsewhere [50]. B1 and B2 small molecule pharmacophores were defined after docking studies of diverse non-peptide selective ligands to each of the two receptors [32,33]. Docking studies were carried out using a set of unique conformations resulted from thorough conformational searches for the diverse ligands studied and rank ordered using the XP score function of GLIDE [51].

## 4. Conclusions

Around 20% of the people infected with SARS-CoV-2 are likely to develop pneumonia of varying severity that may evolve to acute respiratory distress syndrome (ARDS), sepsis, and death. SARS-CoV-2 binds with high affinity to ACE2, mediating cell fusion and entry. ACE2 downregulation was pointed as the origin of the observed inflammatory response of sever cases of Covid-19, mediated by angiotensin II through the AT1 receptor. However, there is an increasing evidence that inflammation is mediated through the bradykinin B1 receptor due to the increased levels of des-Arg^9^BK. Accordingly, antagonists of the bradykinin receptors could be useful therapeutic agents to block the inflammatory signaling process. Presently, icatibant is the only bradykinin antagonist approved drug in the market and there are clinical studies in progress to assess its efficacy for the treatment of Covid-19. However, icatibant is a B2 selective antagonist and it is desirable to have also B1 selective or non-selective antagonists available.

In order to have novel therapeutic treatments available in a short time, drug repurposing is a valuable procedure. Repurposing of already approved drugs has several advantages like their known safety/tolerability profiles, availability and low cost. In order to speed up the identification of approved drugs for novel therapeutic uses, virtual screening can be a valuable tool, provided that structural information on the target is available.

As a continuation of a previous study devoted to identify the molecular features required by compounds to exhibit an antagonist profile to the bradykinin receptors, we discussed in the present manuscript those molecular features that provide a non-selective profile to them. These features were used as a query to carry out a virtual screening on the DrugBank, a database containing all approved drugs. The study yielded eight molecules that were subsequently docked onto the 3D models of the B1 and B2 receptors respectively, to check for possible steric hindrance. Evaluation of their profile as bradykinin antagonists is currently under investigation.
